# Hypnosis for hot flashes among postmenopausal women study: A study protocol of an ongoing randomized clinical trial

**DOI:** 10.1186/1472-6882-11-92

**Published:** 2011-10-11

**Authors:** Gary R Elkins, William I Fisher, Aimee K Johnson

**Affiliations:** 1 Mind-Body Medicine Research Laboratory, Department of Psychology and Neuroscience, One Bear Place # 97334, Baylor University, Waco, TX, USA

## Abstract

**Background:**

Hot flashes are a highly prevalent problem associated with menopause and breast cancer treatments. The recent findings from the Women's Health Initiative have important implications for the significance of a non-hormonal, mind-body intervention for hot flashes in breast cancer survivors. Women who take hormone therapy long-term may have a 1.2 to 2.0 fold increased risk of developing breast cancer. In addition, it is now known that hormone therapy with estrogen and progestin is associated with increased risk of cardiovascular disease and stroke. Currently there are limited options to hormone replacement therapy as non-hormonal pharmacological agents are associated with only modest activity and many adverse side effects. Because of this there is a need for more alternative, non-hormonal therapies. Hypnosis is a mind-body intervention that has been shown to reduce self-reported hot flashes by up to 68% among breast cancer survivors, however, the use of hypnosis for hot flashes among post-menopausal women has not been adequately explored and the efficacy of hypnosis in reducing physiologically measured hot flashes has not yet been determined.

**Methods/design:**

A sample of 180 post-menopausal women will be randomly assigned to either a 5-session Hypnosis Intervention or 5-session structured-attention control with 12 week follow-up. The present study will compare hypnosis to a structured-attention control in reducing hot flashes (perceived and physiologically monitored) in post-menopausal women in a randomized clinical trial. Outcomes will be hot flashes (self-report daily diaries; physiological monitoring; Hot Flash Related Daily Interference Scale), anxiety (State-Trait Anxiety Inventory; Hospital Anxiety and Depression Scale (HADS); anxiety visual analog scale (VAS rating); depression (Center for Epidemiologic Studies Depression Scale), sexual functioning (Sexual Activity Questionnaire), sleep quality (Pittsburgh Sleep Quality Index) and cortisol.

**Discussion:**

This study will be the first full scale test of hypnosis for hot flashes; one of the first studies to examine both perceived impact and physiologically measured impact of a mind-body intervention for hot flashes using state-of-the-art 24 hour ambulatory physiological monitoring; the first study to examine the effect of hypnosis for hot flashes on cortisol; and the first investigation of the role of cognitive expectancies in treatment of hot flashes in comparison to a Structured-Attention Control.

**Trial Registration:**

This clinical trial has been registered with ClinicalTrials.gov, a service of the U.S. National Institutes of Health, ClinicalTrials.gov Identifier: NCT01293695.

## Background

Hot flashes are the most common complaint of post-menopausal women [1,2]. Almost two-thirds of post-menopausal women have hot flashes and nearly 20% find them to be nearly intolerable [3]. It is estimated that over 25 million women in the United States may have hot flashes and 4 million may have severe hot flashes [4]. The experience of hot flashes can be very uncomfortable and can have a very negative impact upon the individual's quality of life [5] energy and sleep [6]. It was also reported that one-third of women with hot flashes described embarrassment and 20% described a general sense of a loss of control [7]. In addition, hot flashes are associated with decreased libido [4]. Hot flashes can significantly decrease quality of life, alter daily activities, and negatively affect sleep [2,8,9] in breast cancer survivors.

The standard of care for ovarian failure and hot flashes is hormone replacement therapy. However, estrogen replacement therapy is a concern for some patients due to an associated increase in the risk of breast cancer. Cancer survivors are contraindicated for estrogen replacement therapy [10]. Recent data from the Women's Health Initiative Trial [11], a randomized controlled trial of hormone therapy with estrogen and progestin as primary prevention in post-menopausal women, suggest that women who take hormone therapy long term have a 1.2 to 2.0 fold increased risk of developing breast cancer. In the Women's Health Initiative Trial, the incidence of breast cancer increased by 26% for women in the estrogen-progestin group. In addition, hormone therapy was shown to be associated with increased cardiovascular disease and stroke. Because of these serious concerns many women reject estrogens [12] and health care providers are reluctant to prescribe it. As a result, there is a need for non-pharmacological interventions for women who suffer from hot flashes.

Because of these concerns about hormonal interventions, efforts have been made to identify non-hormonal agents for hot flashes. Studies have been conducted to investigate the use of: soy supplementation [13] vitamin E [14], gabapentin [15], black cohosh and St. John's wort [16] and clonidine [10,17]. Soy does not seem to be much more effective, or only modestly more effective than placebo [4]. None of these agents have been found to be very efficacious and can be associated with a high toxicity profile [4,18,19].

Other studies have investigated the use of antidepressant medication for hot flashes. In a pilot study of breast cancer survivors, the use of paroxetine hydrochloride (Paxil), showed a mean reduction of hot flashes of 67% [4]. However, adverse reactions to the treatment included somnolence and anxiety in 16% of the participants resulting in discontinuation or reduction in medication. A pilot study to investigate the use of venlafaxine hydrochloride for hot flashes in cancer survivors found that the subjects who completed the study reported a 58% reduction in hot flashes [20]. However, negative effects were found in some participants and included symptoms of depression, dry mouth, fatigue, sleepiness, and difficulty with concentration. More recently, prospective randomized clinical trials have confirmed these findings. Venlafaxine reduced hot flashes by 60% compared to a 20% reduction with placebo [21]. Paroxetine reduced hot flashes by 45-65% in post-menopausal women [22,23]. Similar results were observed in breast cancer survivors [23]. Fluoxetine decreased hot flash frequency by 50% compared to 36% in placebo [24]. Other studies suggested that sertraline was no more effective than placebo in decreasing hot flashes [25].

While these agents are non-hormonal and are not thought to increase the risk of breast cancer they do have side effects that may reduce tolerance for some patients. Also, drug interactions can limit their use and they are not effective for all patients, especially women on anti-estrogen treatment. Importantly, non-hormonal agents have only modest activity in the treatment of hot flashes and have not been found to be as effective as hormonal therapy [19]. Given these facts, it is imperative that effective new interventions be developed to help post-menopausal women who experience hot flashes.

Hypnosis has been demonstrated to be a potentially effective treatment for hot flashes in breast cancer survivors. A study was completed to evaluate the effectiveness of hypnosis for reducing hot flashes among 16 breast cancer survivors [26]. In this study, 16 women with a history of hot flashes demonstrated that a hypnosis intervention could achieve approximately 69% reduction in hot flash scores relative to baseline among breast cancer survivors. In this single arm pilot study we investigated the use of hypnosis to reduce hot flashes [27]. Each patient provided baseline data and received weekly sessions of hypnosis that followed a standardized transcript that allowed for individualization of mental imagery for relaxation and coolness. Throughout the clinical care, participants completed daily diaries of the frequency and severity of their hot flashes. The hot flash score, a measure of severity and frequency, was determined by multiplying the daily frequency of hot flashes by their average severity. Results indicated a 58.6% decrease in total daily hot flashes and a 69.8% decrease in weekly hot flash scores from their baselines. Based upon our initial findings we then completed a second study under the auspices of an R-21 grant. In this study breast cancer survivors with hot flashes were randomized to either a therapist delivered hypnosis intervention or a wait-list control condition. Participants in the hypnosis intervention received 5 sessions of individual hypnosis delivered by a trained hypnotherapist and with individualization of mental imagery for coolness [28]. Results demonstrate that by the end of the treatment period, hot flash scores (frequency × average severity) decreased 68% from baseline to endpoint in the hypnosis arm (*p *< .001). Also, significant improvements in self-reported anxiety, depression, interference of hot flashes on daily activities, and sleep were observed for patients who received the hypnosis intervention (*p *< .005) in comparison to the no-treatment control group.

## Methods/design

### Subjects

A total of approximately 180 post-menopausal women will be entered into the study. A screening checklist will be used to determine eligibility. Participants will be randomly assigned to either a hypnosis intervention or structured-attention control.

### Eligibility Criteria

Eligibility criteria were developed in reference to the Department of Health and Human Services, Food and Drug Administration (FDA) draft recommendations [29], research guidelines [30,31]. Postmenopausal as defined by 1) no menstrual period in the past 12 months; 2) no menstrual period in the past 6 months and a medically documented history of FSH level greater than 40; or 3) women who have had a bilateral oophorectomy. If women have had a hysterectomy and still have their ovaries, they must meet the FSH criteria described above. A participant is eligible if they have a self-reported history of a minimum of 7 hot flashes per day or 50 hot flashes per week at baseline, aged over 18 years and ability to give her own consent for participation in the study, and have discontinued other putative therapies for hot flashes for at least one month prior to enrollment (however, because many of the participants seen in our institutions take vitamin preparations, Vitamin E will be allowed). Further, we participants must be able to attend weekly sessions.

### Exclusion Criteria

Participants will be excluded from participation if they are receiving other simultaneous treatment for hot flashes, or using any CAM (Complementary and Alternative Medicine) treatments for vasomotor symptoms. This would include soy products and other phytoestrogens, black cohosh, and any mind-body techniques including meditation and yoga, etc. Further, participants will be excluded if they have any medical or psychiatric condition that in the opinion of the investigator puts the participant at potential risk during the study, are currently using hypnosis for any reason, or have an inability to speak or understand English.

### Randomization

**Figure 1:**
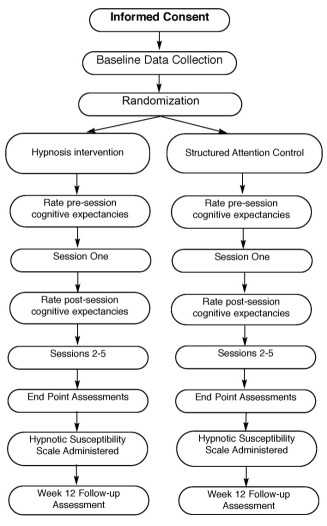
**Study Design**.

After the 7-day baseline data have been collected, participants may return the completed daily diary, Biolog^® ^3991 monitor, Bahr monitor and cortisol samples. Between week 0 and week 1 visits, the Research Coordinator will contact the Data Coordinator who will provide random assignment for the participant, and this assignment will be placed in a sealed envelope that will not be opened until the visit at week 1. Random assignment will be made sequentially from a confidential computer-generated list of permuted blocks of varying size. The list is generated to provide equal probabilities to the study arms. The study participants will be randomly assigned by the Data Coordinator to either the hypnosis intervention or to a structured-attention control group. An overview of the chronology of the study is provided graphically [Figure 1].

### Experimental Intervention

Participants assigned to the hypnosis intervention will be scheduled to meet with a hypnosis research therapist for five visits. All visits will follow eight key components of structured attention guidelines as defined in the treatment manual. In addition, participants in the Hypnosis Intervention will receive a hypnotic induction at each session and a CD recording of a hypnotic induction. The hypnotic induction will follow a well-defined standard transcript but will include mental imagery for relaxation and coolness based on preferences of the participant and encouragement to practice hypnosis on a daily basis with and without the recording.

At the first session the hypnosis research therapist will provide the participant with a handout regarding hot flashes with a hypnosis related description. The use of hypnosis will be discussed and the participant will be provided with information regarding hypnosis. Each participant will be provided with a brief discussion regarding myths and misconceptions about hypnosis and the process used in hypnotic induction [31]. The participants will also be asked to rate their level of anxiety at the beginning of the session and after the hypnotic induction. The research therapist will review a checklist, contained in the treatment manual at each treatment session to ensure consistency.

Participants will be scheduled to return on an approximately weekly basis for sessions 2-5. Each session will include a hypnotic induction and during session 3, a handout with step-by-step instructions in self-hypnosis will be presented. The research therapist will ask the participants about their use of self-hypnosis and their preferences of self-hypnosis (i.e., with the self-hypnosis recording or without using the recording). The research therapist will also discuss the frequency and severity of the participants' hot flashes, and inquire about the effectiveness of self-hypnosis in decreasing the frequency and severity of the hot flashes on a daily basis. Participants will be asked to identify specific imagery for relaxation and imagery for coolness in order to individualize the hypnotic induction based upon the participants' preferences [32].

### Control Intervention

The structured-attention control condition is designed to control for attention, monitoring symptoms, and interpersonal exchange that is inherent in therapeutic hypnosis intervention. In designing our structured-attention control condition we followed the recommendations of Jensen and Patterson [33] that has determined the importance of creating control conditions that include minimal-effect interventions and that are well defined. All visits will follow structured attention guidelines as defined in the treatment manual. The structured attention guidelines will follow eight key components developed specifically for this study. This type of approach (with somewhat different key components for pain control) has previously been used in the study of hypnosis with medical participants [34].

Participants assigned to the structured-attention control condition will meet with a research therapist for five individual approximately weekly visits. The eight key components will be: Information (i.e. causes of hot flashes, triggers for hot flashes); monitoring of hot flashes (i.e. "How many hot flashes did you have yesterday?; "How severe were your hot flashes yesterday?"); rating anxiety (i.e. "Please rate how anxious you feel."); attentive listening; interpersonal exchange; discussion of symptoms (i.e. "How have you been feeling during the past week?": "How have you been sleeping?"); encouragement (i.e. "You are doing well."); and avoidance of negative suggestion. Also, at the first session the research therapist will provide the participant with a handout regarding hot flashes without mention of hypnosis. In addition, participants in the structured-attention control condition will be provided with a CD recording that they can listen to at home. The recording will be approximately the same length of time as the hypnosis recording that those in the hypnosis group will receive, however no hypnotic induction will be provided to the Structured-Attention Control. The research therapist will review a checklist at each intervention session to assure consistency contained in the treatment manual.

### Effect Measures

**Figure 2:**
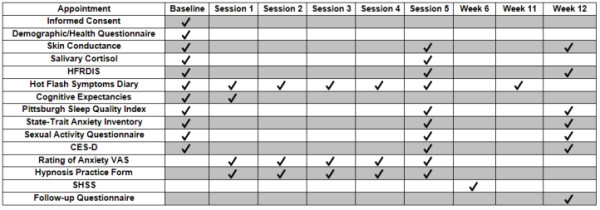
**Data Collection Schedule**.

Outcomes will be hot flashes (self-report daily diaries; physiological monitoring; Hot Flash Related Daily Interference Scale), anxiety (State-Trait Anxiety Inventory; Hospital Anxiety and Depression Scale (HADS); anxiety visual analog scale (VAS rating); depression (Center for Epidemiologic Studies Depression Scale), sexual functioning (Sexual Activity Questionnaire), sleep quality (Pittsburgh Sleep Quality Index) and cortisol; moderators will be cognitive expectancies (VAS ratings) and hypnotizability (Stanford Hypnotic Susceptibility Scale-Form C). A figure representing the schedule of collection is provided [Figure 2].

### Hot Flashes via Sternal Skin Conductance Monitoring

Two different sternal skin conductance monitoring systems may be used to objectively measure hot flash frequency. With the Biolog^® ^ambulatory recorder [35], skin conductance levels are monitored using Medi-Trace silver/silver chloride and a 0.5 constant voltage circuit [36]. Electrodes are 1.5 cm in diameter and filled with 0.05 M potassium chloride Velvachol/glycol gel [37]. Electrodes are attached 1.5" below the collarbones and 2" on either side of the sternal mid-line. Participants will be educated on the proper use of the monitor. The Biolog^® ^monitor is a solid-state device containing a microprocessor and 4 megabytes (MB) of memory. The 3991 was designed by the company with input from the study Consultant, Dr. Carpenter, to provide (1) a smaller and more lightweight hot flash monitor and (2) capability for storing 24 hours' worth of data.

The Bahr Hot Flash Monitor [38] may be used to measure objective hot flashes in conjunction with the Biolog^® ^monitor. The Bahr monitor attaches to a 1" × 2" cloth patch that contains the two electrodes to be placed in the midline over the sternum, two to three inches below the sternoclavicular notch. Prior to connection with the electrodes, the monitor should be synchronized and time stamped with the Bahr watch and software. The monitor should then be activated using a magnet until the green light flashes. The activation time and the attachment time should be recorded in the Monitor Log. When the participant subjectively observes a hot flash, they will be instructed to pass the magnet close to the monitor and a red light will flash to indicate acceptance of the marker. The monitor will measure skin conductance every ten seconds. The participant may wear the monitor for simultaneous comparison with the Biolog^® ^monitor. One advantage of the Bahr monitor is that the dimensions are much smaller than the Biolog^® ^(6 × 2 × 1 cm) and is light enough to simply hang from the chest leads without carrying an extra bag to store the device.

### Salivary cortisol

Salivary cortisol samples will be collected four times in the same day by participants during the week before the first session (baseline) and after completing approximately five sessions of the hypnosis intervention or structured-attention control. Instructions will be provided to participants upon receipt of the cortisol materials. Pre- and post-session salivary cortisol samples will also be collected at the participants' first session with the research therapist. Salivary cortisol samples will be assayed with a time-resolved immunoassay with a cortisolbiotin conjugate as a tracer. Total cortisol produced over a day will be measured by calculating the area-under-the-curve (AUC). This represents the total volume of cortisol secretion over the day. We will also examine cortisol levels at each time point averaged across the areas under the curve and mean diurnal and nocturnal levels will be compared and the presence of circadian rhythmicity will be evaluated [39].

### Statistical power and Sample size

Power analyses were conducted using the partial eta-squared (η_p_^2^) from the repeated measures ANOVAs run for the pilot study. Analyses were based on a sample size of 180 (90 per arm) with a conservative estimate of 30% drop-out. Further, it is recognized that there is the possibility of a placebo effect not observed in the pilot study due to the provision of an active control group. Placebo effects of as large as a 30% reduction in hot flash frequencies and hot flash severity scores have been reported (Sloan et al., 2001). Thus, the calculations reported here are based on a sample size of 126 with effect sizes 30% smaller than those from the pilot study.

An overall experiment-wise alpha of 0.05 will be used, with a Dunn/Bonferroni correction to adjust the error rate for individual analyses. Twenty-one separate analyses are planned (8 for Aim 1, 8 for Aim 2, 5 for Aim 3). Thus, these sample size calculations are based on α = 0.00238 (0.05/21).

Power calculations were conducted using pilot data, analyzed using repeated measures ANOVAs with one between-subjects and one within-subjects variable. Calculations were conducted using G*Power and focused on the interaction term from these analyses. G*Power takes into account the expected effect size (η_p_^2^, reduced by 30%), alpha (0.00238), desired power (0.90), correlation between pre- and post-test, and sphericity. The sample size needed for 0.90 power based on these calculations (CES-D, η_p_^2 ^= 0.0672, *r *= 0.492, pre-post) was 72 (sample size calculations using other dependent variables were smaller). Given a sample size of 180, with a 30% drop out/missing data rate, power would be 0.99 for this comparison. Thus, this study is adequately powered even if a higher than anticipated drop out rate occurs, or if missing data reduces the *N *for some of the analyses.

Participant demographic characteristics will be analyzed using descriptive statistics, including baseline characteristics, averages, and related confidence intervals. Hot flash scores and frequencies will be plotted over the study period. The hot-flash score, being a measure of severity and frequency, is determined by multiplying the daily frequency of hot flashes by their severity (Perez et al., 2004). Analysis will be completed to fully examine and explore the data and to test the hypotheses.

Primary analyses will be conducted using a mixed model approach, with each dependent variable analyzed separately as a pretest-posttest control group design with one within-subjects variable (pre-post) and one between-subjects variable (experimental-control). Composites will be used for analyses with multiple scales (e.g., a single sleep composite will be used).

### Data Quality and Data Monitoring

All data (with the exception of VAS ratings of anxiety, and pre and post measurements of resting heart rate and blood pressure) will be collected by the Research Coordinator and Data Coordinator separate from the therapists providing intervention. The Data Coordinator will oversee data entry. To assure quality, data will be entered into a data base twice by doctoral Students in Psychology at Baylor University or other assigned personnel who will be blinded as to the participants' assignment. The data will then be sent to a biostatistician and checked for consistency and completeness. Any inconsistency or incompleteness will be addressed by the Research Coordinator, Data Coordinator and PI for accuracy. A data safety and monitoring plan has been developed for this study. The P.I. in conjunction with the biostatistician or other assigned personnel will check the data for completeness and quality on a quarterly basis. Once the data has been entered into a data base and data quality has been assured the data base will be subjected to further analyses. As required by NCCAM/NIH, a DSMB will be selected and will periodically review the adverse events and the collected data. Within the 5 year study duration, we expect the DSMB to meet at least annually. The meetings will serve to establish whether the study is accruing appropriately, whether the data collection is running smoothly, whether unexpected toxicities are present, and whether patients are completing both phases of the study. At a minimum, the DSMB will meet at least annually and within 7 days of any serious adverse events.

### Ethical Review

The study protocol ID# 194610-8, "Hypnosis for Hot Flashes Among Postmenopausal Women in a Randomized Clinical Trial" received ethical review from the Baylor University Institutional Review Board and was approved April 1, 2011, signed by Michael E. Sherr, Ph.D., Chair of the Baylor Institutional Review Board, available by phone at 00 1 512 710-4483, or by email at michael_sherr@baylor.edu.

### Current Status of Trial

This trial is currently in accrual at the time this manuscript was prepared. To date, there are no other manuscripts under submission nor published based on this trial.

## Competing interests

The authors declare that they have no competing interests.

## Authors' contributions

GRE is the principal investigator and primary author of this paper. AKJ participated in the construction and maintenance of the data-set and in preparation of this manuscript & WIF participated in the clinical hypnosis intervention and in preparing this manuscript. All authors have read and approved this manuscript.

## Pre-publication history

The pre-publication history for this paper can be accessed here:

http://www.biomedcentral.com/1472-6882/11/92/prepub
